# Relationship between Physical Activity, Screen Time, and Sleep Quantity and Quality in US Adolescents Aged 16–19

**DOI:** 10.3390/ijerph16091524

**Published:** 2019-04-30

**Authors:** Furong Xu, Sue K. Adams, Steven A. Cohen, Jacob E. Earp, Mary L. Greaney

**Affiliations:** 1 Department of Kinesiology, University of Rhode Island, 25 West Independence Way, Kingston, RI 02881, USA; jacob_earp@uri.edu; 2 Department of Human Development and Family Studies, Transition Center, Kingston, RI 02881, USA; suekadams@uri.edu; 3 Health Studies Program, University of Rhode Island, 25 West Independence Way, Kingston, RI 02881, USA; steven_cohen@uri.edu (S.A.C.); mgreaney@uri.edu (M.L.G.)

**Keywords:** physical activity, screen time, sleep quantity, sleep quality, adolescents

## Abstract

Despite the health benefits associated with physical activity (PA), screen time reduction, and sleep quantity and quality, the relationships between PA, screen time, and sleep quantity and quality remain unclear in adolescents. The present study is a cross-sectional analysis of data from adolescents aged 16–19 years who participated in the 2005–2006 National Health and Nutrition Examination Survey (*n* = 542). Multivariable logistic regression models, adjusted for confounders, examined the relationship between objectively measured PA, self-reported screen time, and sleep quantity and quality. Respondents who met the current PA recommendation had 50% lower odds of having sufficient sleep (≥8 h) than those not meeting the recommendation (OR = 0.50, 95% CI: 0.26, 0.94). Respondents who met the screen time recommendation (≤2 h/day) had 55% lower odds of reporting poor sleep quality than those whose screen time exceeded the recommendation (OR = 0.45, 95% CI: 0.22, 0.91), with similar patterns observed for females and males. However, males who met both PA and screen time recommendations had 73% lower odds of reporting poor sleep quality than males who met neither recommendation (OR = 0.27, 95% CI: 0.07, 0.99). In conclusion, PA and screen time are associated with sleep quantity or sleep quality in adolescents, and there are differences in these associations by sex.

## 1. Introduction 

The importance of sleep for health and well-being during adolescence is widely recognized as this is a period of significant growth and development [1,2,3]. However, 45% of adolescents in the United States (US) report insufficient sleep, defined as less than eight hours per night, on school nights [4]. In addition to sleep quantity, adolescents’ sleep quality is concerning [5,6]. A large-scale study of youths in Texas found that 26.8% of respondents aged 11–17 years had insomnia symptoms [5], while another study found that 60.5% of adolescents reported difficulties falling asleep, and 86.8% reported difficulties staying asleep [6]. Insufficient sleep and poor sleep quality can impact adolescents’ school performance, impair emotional regulation, and contribute to a wide range of health conditions, including chronic tiredness or fatigue and increased risk of obesity [7,8,9,10]. Given the importance of the developmental stage of adolescence, it is crucial to ensure that adolescents have adequate and high-quality sleep. 

Despite the compelling evidence that physical activity positively impacts adults’ sleep quantity and quality [11], research examining this relationship in adolescents is lacking, and the few existing studies have had inconsistent findings [12,13,14,15,16,17,18]. Some research with adolescents indicates that meeting the physical activity guidelines (≥60 min/day of moderate-to-vigorous physical activity) is associated with reduced sleep quantity and improved sleep quality [13], while other studies have observed a positive relationship between physical activity and sleep quantity and quality [14,15,16,17,18] or no significant relationship [19]. Moreover, most of these studies have relied on self-reported physical activity and not objectively measured physical activity (e.g., assessed by pedometers or accelerometers) [14,15]. Subjective physical activity measures are less precise and accurate compared with objective measures of physical activity [20]. Given inconsistent study findings and their reliance on self-reported physical activity, it is important to examine this relationship using an objective assessment of physical activity.

Another factor that has been shown to influence sleep is screen time from multiple forms of digital media (e.g., smart phone, computer, tablets, televisions, gaming) [21,22]. Over the last decade, technology has changed at a rapid pace; however, the screen time recommendation for adolescents has remained consistent at 2 h/day or less [21]. Most adolescents (95%) in US now own or have access to smartphones, 51% use some forms of social media (e.g., Facebook, Instagram), and 45% of adolescents report that they are using internet constantly [22]. Problematic technology use transcends both daytime and nighttime making screen time a ubiquitous issue that impacts adolescents’ sleep [22,23]. While many researchers have examined the relationship between screen time and sleep quantity and quality, large-scale studies are lacking, and the results of these studies are difficult to compare due to different measures of screen time, sleep quantity, and sleep quality [23,24,25,26,27,28]. For example, one study found no significant relationship between screen time and sleep quantity [24]. Several studies with adolescents conducted outside of the US have identified an inverse correlation between screen time and sleep quantity, with greater screen time being associated with shorter sleep duration [25,26,27]. Furthermore, a US study found that adolescents who reported multiple forms of screen time use (e.g., smart phone, computer, and television) had decreased sleep quantity [28]. Moreover, research examining the relationship between screen time and sleep quality indicators as outcomes is even sparser. Hysing and colleagues (2014) found that screen time use before bed is associated with increased odds of sleep onset (time needed to fall asleep) latency over 60 min in western Norwegian adolescents [25]. Similarly, a study using data from the 2011 National Sleep Foundation’s Sleep in America Poll found that adolescents (aged 13–21) who used technology (e.g., television, video games, cell phone, and computer) before bed reported inadequate sleep and feeling unrefreshed during the day [23]. Nonetheless, these studies have a small sample size [23,28] and were conducted outside the US [24,25,26,27]. Thus, there is a need for research examining the relationship between screen time and sleep quantity and quality in a nationally representative sample of US adolescents. 

The present study was conducted to examine the relationship between objectively measured physical activity and screen time with both sleep quantity and sleep quality. The primary study aim was to examine the potential associations between physical activity, screen time, and sleep quantity and quality in a nationally representative dataset of US adolescents. The secondary study aim was to explore the combined effect of physical activity and screen time on adolescents’ sleep quantity and quality.

## 2. Material and Methods

This is a cross-sectional analysis of data from the 2005–2006 National Health and Nutrition Examination Survey (NHANES), a publicly available dataset from the Centers for Disease Control and Prevention. The 2005–2006 dataset was used as it is the most recent available NHANES dataset that includes objective measured physical activity and sleep quality data [29]. Of all 2005–2006 NHANES respondents (*N* = 10,348), 5.8% (*n* = 1136) were 16–19 years old at the time of the examination. Of these, 45% (*n* = 542) had physical activity, screen time, and sleep quality data available. Respondents (*n* = 594) who were excluded due to missing data did not influence the representation of the present study. Specifically, no sample characteristic differences were identified between respondents who were included and excluded from the study (see Table S1). Analyses were also guided by Peduzzi and colleagues’ (1996) “one in ten rule” (i.e., one predictive variable can be studied for every 10 events) due to difficulty conducting power calculations for multivariable models. The dataset for the current study has more than 200 events with sleep quantity (≥8 h) and poor sleep quality, allowing for up to 20 predictors to be used in the models with reliable fit [30]. 

The University of Rhode Island Institutional Review Board has determined that this study does not meet the definition of human subject research under the purview of Federal Regulation 45 CFR 46 regarding human subject research, because data used for the current study were de-identified and publicly accessible. 

### 2.1. Physical Activity 

Respondents’ physical activity time was assessed by the ActiGraph 7164 accelerometer (ActiGraph LLC, Fort Walton Beach, FL, USA), which is considered the gold-standard measurement of habitual physical activity time [31,32]. Respondents were asked to wear the accelerometer for seven days during all waking hours except during water activity (e.g., showering, swimming). Accelerometer data were analyzed using the algorithms developed by Troiano and colleagues [33], and a 1-min bout was used to calculate all physical activity time. A valid day was defined as 10+ h of wear time, and only respondents with 4+ days of valid days were included in the analysis [33]. Respondents’ physical activity was categorized into three categories: 1) less active: <30 min/day; 2) active: ≥30 min/day and <60 min/day; 3) very active: ≥60 min/day [34]. Moreover, the US Department of Health and Human Services’ age-specific physical activity recommendations were used to determine if respondents met the physical activity recommendations [35]. That is, respondents aged 16–17 who participated in 60+ min of moderate-to-vigorous physical activity daily and respondents aged 18-19 who participated in 150+ min of moderate intensity physical activity weekly or 75+ min of vigorous intensity physical activity or an equivalent combination of moderate and vigorous physical activity weekly were classified as meeting the physical activity recommendations [35]. 

### 2.2. Screen Time

Respondents reported their average daily TV or videos and computer (h/day) use over the past 30 days [28]. The American Academy of Pediatrics screen time guidelines (≤2 h/day) were used to determine whether respondents met (≤2 h/day) or exceeded (>2 h/day) this recommendation [36].

### 2.3. Sleep Quantity and Quality 

Sleep quantity was assessed by a single item that asked respondents to report how many hours they slept on weekdays, which was then dichotomized to sufficient sleep (≥8 h) or insufficient sleep (<8 h) (4). In addition, sleep quality was assessed by five items; one item assessed the time needed to fall asleep, and four assessed sleep-related problems: frequency of having trouble falling asleep, feeling unrested, being overly sleepy during the day, or not getting enough sleep in the previous 30 days [28]. Poor sleep quality was defined as needing 30+ min to fall asleep or reporting one or more sleep-related problems in the past month [37]. This criterion was modified from Bansil and colleagues’ study (2011) to make it appropriate for adolescents [3,37]. Additionally, a sleep quality index (0–4) was created based on the number of sleep-related problems reported for each respondent, and this score was then dichotomized: (1) zero or one and (2) 2+ sleep-related problems.

### 2.4. Covariates

Potential confounders, which have previously been shown to influence physical activity, screen time, and sleep in adolescents [38,39] were identified from the existing literature [38,39] and incorporated into the directed acyclic graph (see Figure S1). These confounders included age, sex, race/ethnicity (non-Hispanic white, non-Hispanic black, Mexican American, other), parental education (high school diploma or less vs. some college or more), and the ratio of family income to poverty (PIR) [29,38,39]. PIR was calculated by family income and family size and further categorized as at or above (PIR ≥ 1) and below (PIR < 1) the poverty level [40]. All demographic information was collected via household interview or at the mobile examination center (MEC), which is the mobile clinic where NHANES assessments take place [29]. Additionally, body mass index was calculated using height and weight, which was measured by NHANES at the MEC (29).

### 2.5. Data Analysis

The MEC exam 2-year weights were used for all analyses, which is recommended by The National Center for Health Statistics, as the sample weights produce an unbiased national estimate when unequal selection probability is applied, such as the NHANES sample [41]. Sample characteristics are presented as means ± standard error for continuous variables and frequencies and proportions for categorical variables. Characteristics were compared between males and females using PROC SURVEYREG and SURVEYFREQ (CHISQ, based on the Rao–Scott chi-square with an adjusted F statistic). Multivariable logistic regression models were performed using PROC SURVEYLOGISTIC including STRATA, CLUSTER, and WEIGHT statement for the binary outcomes of sleep quantity (≥8 h vs. <8 h) and quality (with vs. without poor sleep quality), and the sleep quality index (2+ vs. 0 or 1 sleep-related problems) to examine the association between physical activity, screen time, and sleep quantity, sleep quality and sleep quality index. In addition, sex, as well as the interaction term, sex multiplied by meeting physical activity or/and screen time recommendations, were added to the models as independent variables to examine whether the association between meeting physical activity or/and screen time recommendations and sleep quantity and sleep quality was modified by sex. Model diagnostics were examined using the survey version of the Hosmer–Lemeshow test, with a two-sided p-value above alpha = 0.05 being viewed as being indicative of a model appropriateness. Additional exploratory analysis examined the sleep quality (% with poor sleep quality) difference between different physical activity levels (less active: <30 min/day; active: ≥30 min/day and <60 min/day; very active: ≥60 min/day) by performing logistic regression models. All models, except exploratory analysis, were adjusted for age, race/ethnicity, parental education level, and PIR [38,39]. Analyses were conducted using SAS version 9.4 (SAS Institute Inc., Cary, NC, USA), and significance was set at *p* < 0.05. Non-statistically significant tendencies were defined as 0.05 < *p* < 0.10.

## 3. Results

Respondents’ mean age was 17.4 ± 0.1 years. The sample was 50.7% female, 41.3% racial/ethnic minorities, 44.1% had a parent with high school education or less, 15.6% were classified as being overweight, and 16.1% had obesity. About one-third (32.6%) of respondents met age specific physical activity recommendations, and 32.3% met screen time recommendations. With respect to sleep, 51.3% had insufficient sleep, 43.8% had poor sleep quality, and 11.1% had two or more sleep-related problems (see Table 1).

**Figure 1:**
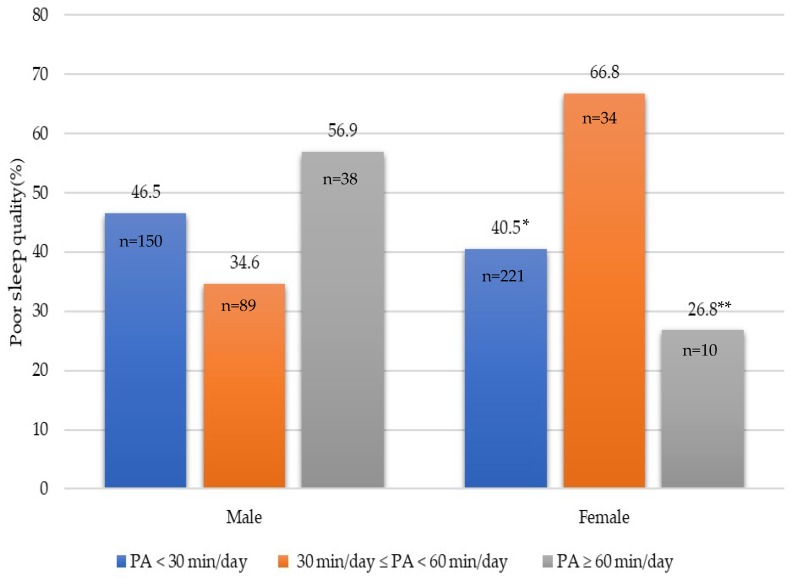
Poor sleep quality (%) and physical activity (PA) levels (min/day) in males (*n* = 277) and females (*n* = 265). Poor sleep quality was defined as needing 30+ min to fall asleep or reporting one or more sleep-related problems in the past month; * PA < 30 min/day different from 30 min/day ≤ PA < 60 min/day, *p* = 0.049; ** PA ≥60 min/day different from 30 min/day ≤ PA < 60 min/day, *p* = 0.003.

Respondents who met the physical activity recommendations had 50% lower odds of reporting sufficient sleep (OR = 0.50, 95% CI: 0.26, 0.94) than those who did not meet the physical activity recommendations. However, the association between meeting the physical activity recommendation and sleep quality was not statistically significant (OR = 0.89, 95% CI: 0.44, 1.80). A similar pattern was observed in females (OR = 0.25, 95% CI: 0.08, 0.77) but not for males (see Table 2). Further exploratory analysis examining the sleep quality differences between different physical activity levels revealed no differences in sleep quality between different physical activity levels. However, among females, 26.8% of those who were very physically active (≥60 min/day) had poor sleep quality compared with 66.8% of those who participated in ≥30 min/day but <60 min/day of physical activity (*p* = 0.003). In addition, there were non-statistically significant tendencies between males who were physically active (≥30 min/day and <60 min/day) and those who were less physically active (<30 min) (34.6% vs. 46.5%, *p* = 0.520) (see Figure 1). 

Screen time was not associated with sleep quantity; however, respondents who met the screen time recommendation (≤2 h/day) had 55% lower odds of having poor sleep quality than those who exceeded the recommendation (OR = 0.45, 95% CI: 0.22, 0.91). Similar patterns were observed among males (OR = 0.44, 95% CI: 0.19, 1.00) and females (OR = 0.44, 95% CI: 0.21, 0.94).

Among respondents who met both the physical activity and screen time recommendations, no significant differences for sleep quantity and sleep quality were found. However, males who met both recommendations had 73% lower odds of having poor sleep quality than those who met neither recommendation (OR = 0.27, 95% CI: 0.07, 0.99). For females, although no significant associations were observed, it is worth noting that there were non-statistically significant tendencies for females who met both physical activity and screen time recommendations (OR = 0.10, 95% CI: 0.01, 1.33) (see Table 2). 

## 4. Discussion

The present study examined the associations between physical activity, screen time, and sleep quantity and quality. Study results indicate that physical activity and screen time are associated with adolescents’ sleep quality and highlights the importance of promoting physical activity and reducing screen time to improve sleep quality among adolescents. 

The current study found that adolescents who met the physical activity recommendation were more likely to have insufficient sleep (<8 h/day) compared with those who did not meet this recommendation. Relatively few studies have examined the relationship between physical activity and sleep in adolescents using objectively measured physical activity, and existing study findings are inconsistent [13,16,19]. Similar to the current study, Olds and colleagues (2011) found that physical activity was associated with shorter sleep duration in Australian youth aged 9–16 years [13]. Other studies have either found that physical activity was positively associated with sleep quantity [16] or that there was no significant association [19]. Comparisons with the current study should be made with caution due to the use of different sleep quantity measures (e.g., continuous vs. dichotomous) [13], different cut points used to define sufficient sleep (e.g., 8 h vs. 9–10 h) [16,19], and adjusting for different confounders [13,16,19] and different geographical populations (e.g., other countries) [13,16,19]. Additionally, the present study found no significant association between physical activity and sleep quality, which contrasts with findings from other larger scale studies that identified a positive association between physical activity and sleep quality [17,18,42]. This difference may be due to different physical activity assessments as the present study used an objective measure of physical activity, while the other studies relied on self-reported physical activity. 

Moreover, this is the first large-scale study examining physical activity and sleep quality that has used an objective measure of physical activity. It is possible that the lack of an observed relationship between meeting the physical activity recommendation and sleep quality in the present study can be explained by the idea that certain physical activity levels may be associated with better sleep quality. This idea supported by the results of our exploratory analysis (females: 60+ min/day, males: ≥30 min/day and <60 min/day). This finding suggests that it may be overly simplistic to deduce that more physical activity time predicts better sleep quality. Instead, the appropriate amount of physical activity might be more important for sleep quality, and sex differences need to be considered as well. Future research should investigate the relationship between physical activity levels and sleep in a representative sample of adolescents given the complexity of sleep [14,42]. 

Study results also indicate that meeting screen time recommendations was associated with better sleep quality overall, although no significant association between screen time and sleep quantity was observed. Adolescents who use technology before bed may go to bed later due to being overly engaged in technology, resulting in decreased sleep quantity [23,43]. Our findings did not confirm this pattern, but they are consistent with other studies that have found that elevated technology use results in poor sleep quality [25,44,45,46]. Moreover, adolescents may have experienced increased cognitive stimulation due to elevated TV and computer exposure before bed [44]. This, along with blue light exposure, can result in physiological changes that impede sleep, including increased sympathetic nervous system response and decreased melatonin secretion [45]. Alternatively, high technology use, especially video gaming, can stimulate a fight or flight response during sleep, resulting in less time spent in stages 3 and 4 of sleep and decreased sleep quality [46]. Moreover, the present study found that both males and females who meet screen time recommendations had better sleep quality, but only females who meet screen time recommendations were more likely to have insufficient sleep. These results support the possibility that for males, the types of screen time that they are engaged in and the timing of engagement may differentially impact their sleep quality [47]. Regardless of the challenge to compare the present study to previous studies due to different confounders [15,23,25,26,27,28] and sample populations [25,26,27], our findings help to elucidate the inconsistent findings in studies that have examined the relationship between screen time and sleep quantity and quality in adolescents [15,23,25,26,27,28].

In addition, we found that males who met both physical activity and screen time recommendations were more likely to have good sleep quality than those who met neither recommendation. This pattern was not observed in females; however, the likelihood of having two or more sleep-related problems was greater for females who met neither the physical nor screen time recommendations than those who met both recommendations. This suggests that underlying sleep-related issues may exist and that they were not uncovered via measures of sleep quality and sleep quantity. The nonsignificant relationship could also be due to the small subgroup sample size (*n* = 3). This is the first study, to our knowledge, that examined the association between combined physical activity and screen time behavior and sleep quantity or sleep quality in a representative sample of US adolescents using objectively measured physical activity. This finding addressed the benefits of physical activity and limited screen time for adolescents’ sleep quality, but the sex difference observed might be due to males and females responding to physical activity differently.

The study findings, however, should be considered in light of the study’s strengths and limitations. The study’s strengths include the use of nationally representative data with a racially (41.3% minority) and socioeconomically (17.5% with a family income below the federal poverty level, 44.1% of parents with high school education or less) diverse sample. An additional strength is the use of objectively measured physical activity and analyses that adjusted for possible individual confounding factors, such as age, race/ethnicity, body mass index, parent education and family income. Furthermore, the cutoff point used for sufficient (≥8 h/day) and insufficient sleep (<8 h/day) was based on the National Sleep Foundation recommendations for specific age groups [4], which can be easily interpreted, compared, or adopted by health practitioners. The study’s limitations include the cross-sectional study design, which does not allow for causal links to be established. Moreover, sleep quantity and sleep quality were assessed by self-report, although this is common in sleep-related studies in adolescents [15]. Additionally, the present study was not designed to assess the timing or the type of physical activity, although previous studies indicate that this might impact the timing of circadian system and sleep quantity in adolescent athletes or in children [48,49,50]. Additionally, there may be residual confounding limitations as some confounders were not measured or available for analysis in the analytic dataset (e.g., anxiety, depression) [51]. However, this is common for studies, such as ours, that utilize secondary sources of data [52]. We were only able to use one data cycle due to the available data for objective physical activity and sleep quality that limited our ability to run subgroup analyses. Lastly, the data used for this study were over 10 years old at the time of analysis, but it is the most recent NHANES dataset available having both objective physical activity and sleep quality measures. This is important to note as screen time usage across all devices has also increased since the time of data collection. 

## 5. Conclusions

In conclusion, adolescents meeting physical activity recommendations may be more likely to experience insufficient sleep but not impaired sleep quality compared with those who met the physical activity recommendations. However, certain physical activity levels may be associated with better sleep quality among adolescents, with the optimal amount of physical activity varying in males and females. Meeting the screen time recommendations was associated with better sleep quality but not with sleep quantity. Furthermore, for males, meeting both physical activity and screen time recommendations were positively associated with sleep quality. Study findings support that physical activity promotion and screen time reduction are important strategies to improve sleep quality in adolescents. Beyond the variables studied in the present study (e.g., age, sex, meeting physical activity and screen time recommendations, etc.), future research can explore the impact of different aspects of physical activity (e.g., amount, intensity level, type of physical activity, physical activity timing during the day) and screen time (e.g., current patterns and types of technology) on adolescents’ sleep.

## Figures and Tables

**Table 1:** Respondents’ characteristics by sex, 2005–2006 National Health and Nutrition Examination Survey (NHANES) (*N* = 542).

Variables	Total	Male	Female	*p*-Value
	*N* = 542	*n* = 277 (49.3%)	*n* = 265 (50.7%)	
Age (yrs)	17.4 ± 0.1	17.5 ± 0.1	17.3 ± 0.1	0.111
Race/ethnicity				
Non-Hispanic White (n%)	123 (58.7)	61 (59.6)	62 (57.9)	0.594
Non-Hispanic black (n%)	202 (17.4)	107 (18.4)	95 (16.5)	
Mexican American (n%)	174 (11.6)	88 (12.2)	86 (11.0)	
Others (n%)	43 (12.3)	21 (9.8)	22 (14.6)	
Parent education				
High school or less (n%)	280 (44.1)	149 (49.0)	131 (39.2)	0.194
College or above (n%)	235 (55.9)	115 (51.0)	120 (60.8)	
PIR				
PIR below poverty <1 (n%)	156 (17.5)	82 (20.6)	74 (14.4)	0.186
PIR at or above poverty ≥1 (n%)	365 (82.5)	184 (79.4)	181 (85.6)	
Body Mass Index (kg/m^2^)	24.2 ± 0.4	24.5 ± 0.5	23.9 ± 0.5	0.318
Underweight ^&^ (n%)	14 (2.8)	7 (2.8)	7 (2.8)	0.645
Normal or Healthy Weight (n%)	338 (65.5)	174 (62.2)	164 (68.8)	
Overweight (n%)	86 (15.6)	43 (16.3)	43 (14.8)	
Obese (n%)	103 (16.1)	52 (18.8)	51 (13.6)	
Sleep quantity (h)	7.3 ± 0.1	7.3 ± 0.1	7.3 ± 0.2	0.738
Sufficient sleep ^@^ (n%)	264 (48.7)	133 (48.3)	131 (49.2)	0.9
Poor sleep quality (n%)	238 (43.8)	119 (44.3)	119 (43.4)	0.886
Poor SQI				
SQI = 0 (n%)	304 (56.2)	158 (55.7)	146 (56.6)	0.286
SQI = 1 (n%)	178 (32.7)	98 (35.5)	80 (30.0)	
SQI = 2+ (n%)	59 (11.1)	21 (8.8)	38 (13.4)	
Total physical activity (min/day)	24.7 ± 1.6	32.5 ± 2.5	17.1 ± 1.9	<0.001*
Meet PA recommendation ^#^ (n%)	187 (32.6)	127 (44.7)	60 (20.7)	<0.001*
Less active <30 min/day (n%)	371 (70.7)	150 (56.9)	221 (84.2)	<0.001*
Active ≥30 and <60 min/day (n%)	123 (21.4)	89 (30.2)	34 (12.8)	0.003*
Very active ≥ 60 min/day (n%)	48 (7.9)	38 (13.0)	10 (2.9)	<0.001*
Screen time (h/day)	4.6 ± 0.2	5.0 ± 0.2	4.1 ± 0.3	0.017*
Meet screen time recommendation ^$^ (n%)	131 (32.3)	54 (28.0)	77 (36.5)	0.097
Meet both PA and screen time recommendation (n%)	34 (8.6)	18 (9.2)	16 (8.1)	0.725

*Note:* means ± standard error for continuous variables, and frequencies and proportions for categorical variables; *p*-values are from PROC SURVEYREG and SURVEYFREQ (CHISQ, based on the Rao–Scott chi-square with an adjusted F statistic). * *p* < 0.05; PIR = family income to poverty ration, PA = physical activity, SQI = sleep quality index; ^&^ underweight <5th percentile, normal or healthy weight ≥5th percentile and <85th percentile, overweight ≥85th and <95th percentile, obese ≥95th percentile; ^@^ sleep quantity ≥8 h; ^#^ 60+ min/day PA for respondents aged 16–17 and 150+ min/week moderate intensity PA or 75+ min/week vigorous intensity physical or an equivalent combination of moderate and vigorous PA for respondents aged 18–19; ^$^ screen time ≤ 2 h/day.

**Table 2:** The association between physical activity, screen time, and sleep quantity and sleep quality, 2005–2006 NHANES (*N* = 542).

Total	Sufficient Sleep ^@^		Classified as Poor Sleep Quality		Sleep Quality Index (2+ Sleep-Related Problems)	
	Adjusted OR (95% CI)	*p* Value	Adjusted OR (95% CI)	*p* Value	Adjusted OR (95% CI)	*p* Value
Meet PA recommendation ^#^	0.50 (0.26, 0.94)	0.033 *	0.89 (0.44, 1.80)	0.727	1.10 (0.40, 3.04)	0.848
P values for meeting PA recommendation * sex	0.472		0.459		0.474	
Meet screen time recommendation ^$^	0.75 (0.45, 1.26)	0.256	0.45 (0.22, 0.91)	0.028 *	0.60 (0.18, 2.05)	0.393
P values for meeting screen time recommendation * sex	0.027*		0.793		0.757	
Meet both PA and screen time recommendation	0.64 (0.22, 1.86)	0.387	0.57 (0.25, 1.30)	0.163	0.42 (0.06, 3.11)	0.373
*p* values for meeting both * sex	0.836		0.043*		0.192	
Males						
Meet PA recommendation ^#^	0.90 (0.42, 1.93)	0.776	0.73 (0.26, 2.07)	0.529	0.81 (0.16, 4.09)	0.784
Meet Screen time recommendation ^$^	1.03 (0.54, 1.95)	0.933	0.44 (0.19, 1.00)	0.046 *	0.81 (0.12, 5.26)	0.812
Meet both PA and screen time recommendation	0.76 (0.23, 2.52)	0.630	0.27 (0.07, 0.99)	0.048 *	1.15 (0.10, 13.32)	0.903
Females						
Meet PA recommendation ^#^	0.25 (0.08, 0.77)	0.019 *	1.19 (0.48, 2.96)	0.692	1.72 (0.55, 5.37)	0.329
Meet screen time recommendation ^$^	0.48 (0.24, 0.96)	0.038 *	0.44 (0.21, 0.94)	0.036 *	0.53 (0.11, 2.61)	0.412
Meet both PA and screen time recommendation	0.46 (0.08, 2.66)	0.357	1.15 (0.38, 3.49)	0.791	0.10 (0.01, 1.33)	0.078

*Note*: adjusted all demographic variables including age, race/ethnicity, parent education, family income-to-poverty ratio, body mass index; ORs, 95% CIs, and *p*-values are from PROC SURVEYLOGISTIC including STRATA, CLUSTER, and WEIGHT statement; * *p* < 0.05; PA = physical activity; ^@^ sleep quantity ≥8 h; ^#^ 60+ min/day physical activity for respondents aged 16–17 and 150+ min/week moderate intensity physical activity or 75+ min/week vigorous intensity physical or an equivalent combination of moderate and vigorous physical activity for respondents aged 18–19; ^$^ screen time recommendation ≤2 h/day.

## Supplementary Materials

The following are available online at https://www.mdpi.com/1660-4601/16/9/1524/s1, Table S1: Subjects’ characteristics for those who have data compared with those who do not have completed data, NHANES 2005–2006, Figure S1: The directed acyclic graph to illustrate the association between covariates and primary exposure and outcomes.
