# Supplementary material for: Relationship between Physical Activity, Screen Time, and Sleep Quantity and Quality in US Adolescents Aged 16–19
Source: Int J Environ Res Public Health. 2019 Apr 30;16(9):1524. doi: 10.3390/ijerph16091524 (PMC6539318; doi:10.3390/ijerph16091524)

Supplementary Table 1. Subjects characteristics for those who have data to those who don't have completed data, NHANES 2005-2006

Variables	Total	With completed data	Without completed data	P-value
	N=1136	n=542 (45%)	n=594 (55%)	
Age (yrs)	17.4 ±0.1	17.4 ±0.1	17.4 ±0.1	0.575
Race/ethnicity				
Non-Hispanic White (n%)	302 (63.5)	123 (58.7)	179 (67.3)	0.198
Non-Hispanic black (n%)	395 (15.5)	202 (17.4)	193 (13.8)	
Mexican American (n%)	353 (10.5)	174 (11.6)	179 (9.7)	
Others (n%)	86 (10.6)	43 (12.3)	43 (9.2)	
Parent education				
High school or less (n%)	604 (47.2)	280 (44.1)	324 (50.0)	0.068
College or above (n%)	453 (52.8)	235 (55.9)	218 (50.0)	
PIR				
PIR below poverty <1 (n%)	340 (18.8)	156 (17.5)	184 (19.9)	0.369
PIR at or above poverty ≥1 (n%)	724 (81.2)	365 (82.5)	359 (80.1)	
Body Mass Index (kg/m^2)	24.6 ±0.3	24.2 ±0.4	24.9 ±0.4	0.139
Underweight[&] (n%)	34 (4.0)	14 (2.8)	20 (5.0)	0.329
Normal or Healthy (n%)	656 (62.4)	338 (65.5)	318 (59.8)	
Overweight (n%)	196 (15.5)	86 (15.6)	110 (15.5)	
Obese (n%)	244 (18.1)	103 (16.1)	141 (19.6)	

Note: means ±standard error for continuous variables and frequencies and proportions for categorical variables; p-values are from PROC SURVEYREG and SURVEYFREQ (CHISQ, based on the Rao-Scott chi-square with an adjusted F statistic); *p<0.05; PIR=family income to poverty ratio; & underweight < 5th percentile, normal or healthy weight ≥5th percentile and <85th percentile, overweight ≥85th and <95th percentile, obese ≥95th percentile.

Supplementary Figure 1. The directed acyclic graph to illustrate the association between covariates and primary exposure and outcomes

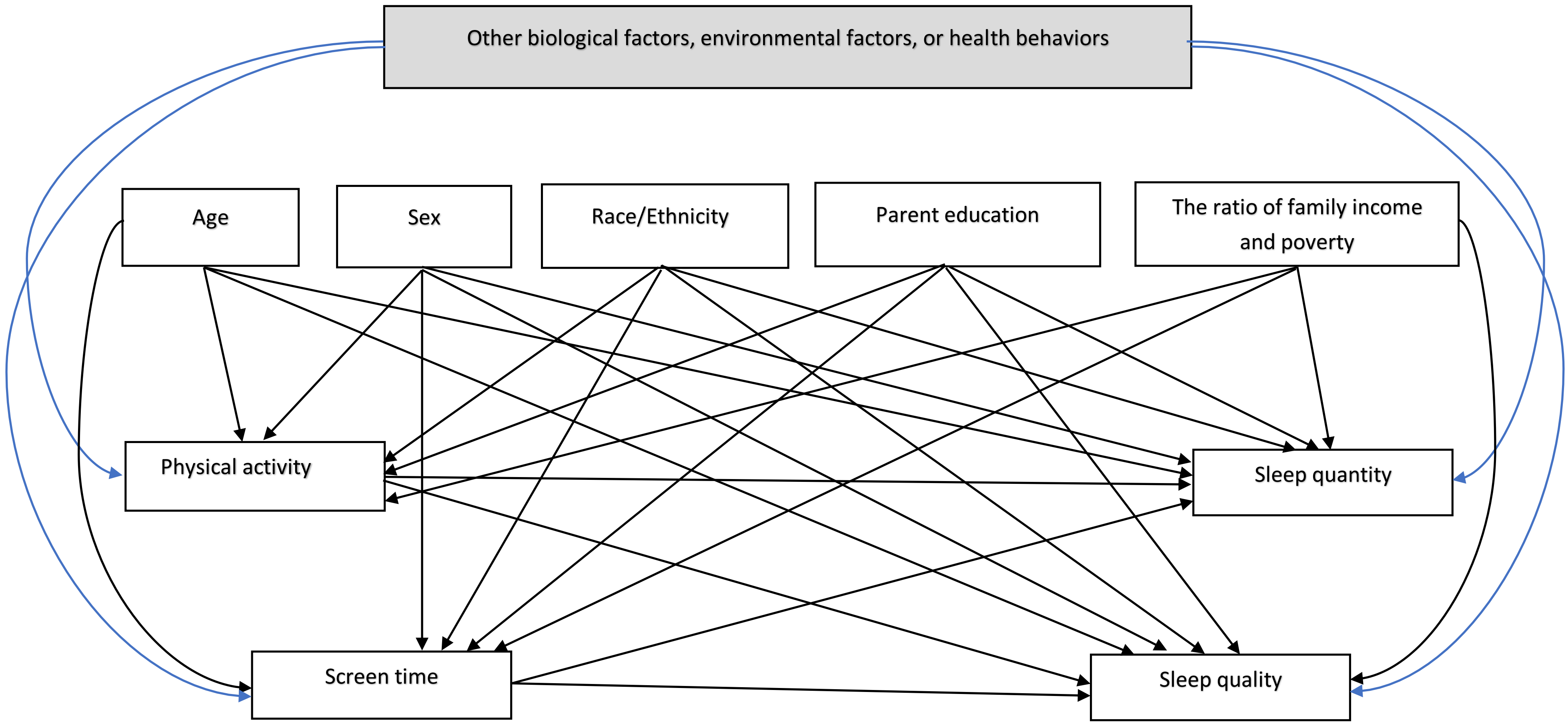

Supplement: Supplementary file 1 [file ijerph-16-01524-s001.pdf]
